# Strategies Used by Healthy Kids, Healthy Communities Partnerships to Prevent Childhood Obesity

**Published:** 2011-12-15

**Authors:** Punam Ohri-Vachaspati, Laura Leviton, Philip Bors, Sarah Strunk, Laura Brennan, Ross C. Brownson

**Affiliations:** School of Nutrition and Health Promotion, Arizona State University; Robert Wood Johnson Foundation, Princeton, New Jersey; Gillings School of Global Public Health, University of North Carolina at Chapel Hill, Chapel Hill, North Carolina; Gillings School of Global Public Health, University of North Carolina at Chapel Hill, Chapel Hill, North Carolina; Transtria, LLC, St. Louis, Missouri; Prevention Research Center in St. Louis. Dr Brownson is also affiliated with the George Warren Brown School of Social Work, the Department of Surgery, and the Alvin J. Siteman Cancer Center, Washington University in St. Louis, St. Louis, Missouri

## Abstract

**Introduction:**

Healthy Kids, Healthy Communities (HKHC) is an initiative of the Robert Wood Johnson Foundation to prevent obesity among high-risk children by changing local policies, systems, and environments. In 2009, 105 community partnerships applied for funding from HKHC. Later that year, the Centers for Disease Control and Prevention (CDC) released recommended community strategies to prevent obesity by changing environments and policies. The objective of this analysis was to describe the strategies proposed by the 41 HKHC partnerships that received funding and compare them to the CDC recommendations.

**Methods:**

We analyzed the funded proposals to assess the types and prevalence of the strategies proposed and mapped them onto the CDC recommendations.

**Results:**

The most prevalent strategies proposed by HKHC-funded partnerships were providing incentives to retailers to locate and serve healthier foods in underserved areas, improving mechanisms for purchasing food from farms, enhancing infrastructure that supports walking and cycling, and improving access to outdoor recreational facilities.

**Conclusion:**

The strategies proposed by HKHC partnerships were well aligned with the CDC recommendations. The popular strategies proposed by HKHC partnerships were those for which there were existing examples of successful implementation. Our analysis provides an example of how information from communities, obtained through grant-writing efforts, can be used to assess the status of the field, guide future research, and provide direction for future investments.

## Introduction

**Box.** CDC-Recommended Community Strategies and Measurements for Preventing Obesity in the United States, 2009^a^

**Category 1: Strategies to promote the availability of affordable healthy food and beverages**
1: Communities should increase availability of healthier food and beverage choices in public service venues.
2: Communities should improve availability of affordable healthier food and beverage choices in public service venues.
3: Communities should improve geographic availability of supermarkets in underserved areas.
4: Communities should provide incentives to food retailers to locate in and/or offer healthier food and beverage choices in underserved areas.
5: Communities should improve availability of mechanisms for purchasing foods from farms.
6: Communities should provide incentives for the production, distribution, and procurement of foods from local farms.
**Category 2: Strategies to support healthy food and beverage choices**
7: Communities should restrict availability of less healthy foods and beverages in public service venues.
8: Communities should institute smaller portion size options in public service venues.
9: Communities should limit advertisements of less healthy foods and beverages.
10: Communities should discourage consumption of sugar-sweetened beverages.
**Category 3: Strategy to encourage breastfeeding**
11: Communities should increase support for breastfeeding.
**Category 4: Strategies to encourage physical activity or limit sedentary activity among children and youth**
12: Communities should require physical education (PE) in schools.
13: Communities should increase the amount of physical activity in PE programs in schools.
14: Communities should increase opportunities for extracurricular physical activity.
15: Communities should reduce screen time in public service venues.
**Category 5: Strategies to create safe communities that support physical activity**
16: Communities should improve access to outdoor recreational facilities.
17: Communities should enhance infrastructure supporting cycling.
18: Communities should enhance infrastructure supporting walking.
19: Communities should support locating schools within easy walking distance of residential areas.
20: Communities should improve access to public transportation.
21: Communities should zone for mixed-use development.
22: Communities should enhance personal safety in areas where people are or could be physically active.
23: Communities should enhance traffic safety in areas where people are or could be physically active.
**Category 6: Strategy to encourage communities to organize for change**
24: Communities should participate in community coalitions or partnerships to address obesity.

Abbreviation: CDC, Centers for Disease Control and Prevention.

a Source: Khan et al (3).

The Centers for Disease Control and Prevention (CDC) and the Institute of Medicine (IOM) promote adding environmental and policy changes to the existing methods of health promotion to prevent obesity (1,2), and they have recently recommended evidence-based strategies for preventing obesity in the United States (3,4). The Robert Wood Johnson Foundation's (RWJF) Healthy Kids, Healthy Communities (HKHC) national program aims to support healthier communities by implementing healthy eating and active living policies, systems, and environmental changes (5). The program focuses on reaching children at highest risk for obesity because of their race/ethnicity, income, or location. Full proposals for the second round of the HKHC initiative were submitted in May 2009 after the launch of a limited number of sites in the first round in 2008. The HKHC program proposals allow a snapshot of the strategies the communities selected to address childhood obesity. We compared these proposed strategies with expert recommendations. The CDC and IOM recommendations for preventing obesity overlap (3,4), but we focus only on the strategies released by CDC in July 2009, soon after the submission of the HKHC proposals. Drawing on the best available evidence and expert opinion, the CDC recommendations, developed for local governments and communities, include a set of 24 community strategies grouped into 6 categories (Box) (3).

The objectives of this research were to undertake a content analysis of 41 funded HKHC proposals and compare them with the CDC recommendations to better understand 1) how the HKHC proposed strategies aligned with CDC recommendations that came later, 2) which strategies were proposed by funded HKHC communities with different characteristics, 3) which strategies were proposed by communities despite limited information about effectiveness, and 4) the relationship between broad national recommendations and concrete local community strategies.

## Methods

RWJF received 540 brief proposals for the second round of HKHC funding in February 2009, after launching 9 leading sites in December 2008. From this pool, 110 community partnerships were invited to submit full proposals. Finally, 41 grantees were selected from 105 full proposal submissions based on guidelines laid out in the call for proposals and on reviewers' predetermined criteria. Reviewers included RWJF staff, HKHC staff, members of the National Advisory Committee for the HKHC initiative, and other external representatives with expertise in childhood obesity prevention or community-based initiatives. Each review committee had at least 1 representative from each group. Communities were considered high-priority if they had a high representation of children from low-income households, had a high percentage of racial/ethnic minority groups at higher risk of obesity, and were in states with a high prevalence of childhood obesity (Alabama, Arkansas, Arizona, Florida, Georgia, Kentucky, Louisiana, Mississippi, New Mexico, North Carolina, Oklahoma, Tennessee, Texas, South Carolina, and West Virginia).

HKHC defined a community as a municipality, county, or region. The HKHC call for proposals focused on approaches that involved policy, systems, and environmental changes for preventing childhood obesity in settings outside the school day. Although the community partnerships were to propose their best thinking on the types of changes they might pursue, the grant program included a planning period during which communities conducted assessments and received technical assistance from the HKHC National Program Office to refine their strategies to meet their community's needs. The HKHC call for proposals was developed independently from the CDC recommendations, despite RWJF involvement in both. The technical assistance provided to applicants focused on guiding them toward selecting approaches that focused on policy, systems, and environmental changes and not on selecting specific strategies.

Proposals were submitted to RWJF using an online submission system. Funding decisions were based on need, apparent capacity, feasibility of implementation, and geographic and demographic diversity. The detailed content analysis described in this article was limited to full proposals submitted by the 41 HKHC grantees.

We analyzed the content of proposals in a 3-step process that used standardized abstraction and analysis forms. In Step 1, information from the proposal narrative was abstracted into Excel spreadsheets and classified according to the following themes: organizational capacity (description, mission, and unique characteristics); community partnerships (lead agency background, age of partnerships, key partners, previous work); proposed initiative (plan, evidence used, goals, major activities, and outcomes); and readiness (previous assessments, support from decision makers and politicians). Variables describing the communities and partnerships were quantified from the abstracted information. A more detailed analysis conducted on abstracted sections described communities' proposed plans and strategies to map them onto the CDC recommendations. In Step 2, the proposed HKHC strategies were sorted into the 6 broad categories of CDC recommendations described in the box. In Step 3, each proposed strategy was matched with the corresponding CDC recommendation in the identified category. For some, information was available only for broad initiatives from the community; such cases were classified as "strategy to be determinedd" under the appropriate category. The research staff conducted the initial proposal abstraction (Step 1) under the supervision of one of the authors (L.B.). The lead author (P.O.V.) mapped and matched proposed strategies (Steps 2 and 3) with help from a trained research assistant; they both mapped the proposed strategies onto the CDC recommendations, and in cases of disagreement a resolution was made in consultation with another author (L.C.L.).

Most of the community strategies proposed were highly consistent with the CDC recommendations and with CDC's suggested indicators to measure progress over time. If a community strategy matched the general aim of a CDC recommendation but was not consistent with the proposed CDC indicator of progress, it was classified as a match with the CDC recommendation. For example, CDC recommendation 18 is to enhance infrastructure to support walking. This recommendation can include a variety of efforts to promote walking; however, the suggested indicator of progress quantifies only paved sidewalk miles in the jurisdiction. HKHC communities chose various strategies to enhance walking, in addition to paving new sidewalks: signalized intersections, signage, and traffic-calming measures. All these strategies were classified as consistent with the CDC recommendation 18.

Although CDC used 6 general categories of recommendations, we analyzed only 4 in the analysis. Given HKHC's focus on children aged 3 to 18 years, communities did not propose to encourage breastfeeding (CDC category 3). Also, because the HKHC call for proposals required communities to work through multidisciplinary partnerships (CDC category 6), all communities had established partnerships, and no variability in assessment was anticipated.

Not all of the community strategies fit the CDC recommendations. Nevertheless, all community strategies were abstracted to capture the HKHC partnerships' understanding of the possible approaches to policy, systems, and environmental change. These strategies were categorized as community gardens, food policy councils, health promotion, and education.

## Results

Twenty-three of the funded communities were from the priority states (Table 1). All but 2 had a median income below $50,000, more than half exceeded 100,000 population, and more than half were in urban areas. Almost half of the partnerships in the funded communities had 3 or more years of experience working together.

Of the 41 proposals selected for funding, 12 community partnerships proposed to work at the county level, 21 at the municipal level, and 5 at the regional level. In most cases, leadership for these proposals was provided by nonprofit organizations (19 sites) and government departments (14 sites) (Table 2).

### Matching HKHC proposed strategies with CDC recommendations

**CDC category 1: strategies to promote the availability of affordable healthy food and beverages**

Thirty-one partnerships proposed at least 1 strategy to promote the availability of affordable healthy foods and beverages (Table 2). Providing incentives to food retailers and improving mechanisms for purchasing foods from farms were proposed by almost half of the partnerships (Figure). Incentives to food retailers included targeted economic development loans for small stores and technical assistance for creating links with federal food assistance programs. Strategies for improving mechanisms for purchasing foods from farms included establishing new farmers' markets; improving their capabilities for using the electronic benefits transfer cards and vouchers from the Special Supplemental Nutrition Program for Women, Infants and Children; and expanding transportation options.

**Figure.**
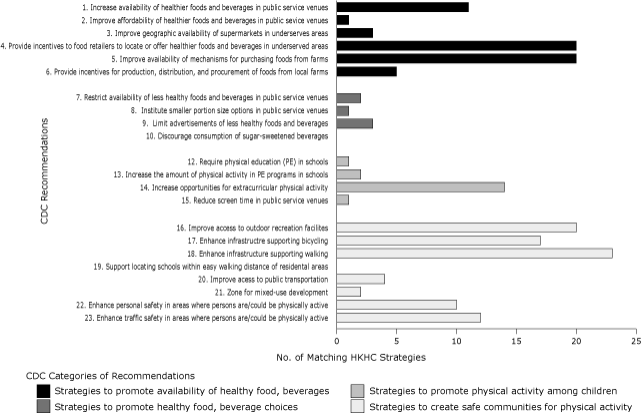
Number of childhood obesity prevention strategies proposed by the 41 Robert Wood Johnson Foundation's Healthy Kids, Healthy Communities funded sites and classified by Centers for Disease Control and Prevention (CDC) as recommendations for preventing obesity. Each site could propose more than 1 strategy; in addition, 21 strategies that were too broad to match the CDC recommendations were classified as "to be determined."

**Table:** 

**CDC Category**	**CDC Recommendations**	**No. of Matching Strategies Proposed by HKHC Sites**
**Strategies to promote the availability of affordable healthy food and beverages**	1: Communities should increase availability of healthier food and beverage choices in public service venues.	11
	2: Communities should improve availability of affordable healthier food and beverage choices in public service venues.	1
	3: Communities should improve geographic availability of supermarkets in underserved areas.	3
	4: Communities should provide incentives to food retailers to locate in and/or offer healthier food and beverage choices in underserved areas.	20
	5: Communities should improve availability of mechanisms for purchasing foods from farms.	20
	6: Communities should provide incentives for the production, distribution, and procurement of foods from local farms.	5

**Strategies to support healthy food and beverage choices**	7: Communities should restrict availability of less healthy foods and beverages in public service venues.	2
	8: Communities should institute smaller portion size options in public service venues.	1
	9: Communities should limit advertisements of less healthy foods and beverages.	3
	10: Communities should discourage consumption of sugar-sweetened beverages.	0

**Strategies to encourage physical activity or limit sedentary activity among children and youth**	12: Communities should require physical education (PE) in schools.	1
	13: Communities should increase the amount of physical activity in PE programs in schools.	2
	14: Communities should increase opportunities for extracurricular physical activity.	14
	15: Communities should reduce screen time in public service venues.	1

**Strategies to create safe communities that support physical activity**	16: Communities should improve access to outdoor recreational facilities.	20
	17: Communities should enhance infrastructure supporting cycling.	17
	18: Communities should enhance infrastructure supporting walking.	23
	19: Communities should support locating schools within easy walking distance of residential areas.	0
	20: Communities should improve access to public transportation.	4
	21: Communities should zone for mixed-use development.	2
	22: Communities should enhance personal safety in areas where people are or could be physically active.	10
	23: Communities should enhance traffic safety in areas where people are or could be physically active.	12

Eleven of the partnerships also proposed increasing the availability of healthier foods and beverages in public service venues, including day care and after-school settings and parks.

**CDC category 2: strategies to support healthy food and beverage choices**

Only 4 partnerships proposed strategies for supporting healthy food and beverage choices (Table 2). Limiting advertising for less healthy food, restricting availability of unhealthy choices, and instituting smaller portion sizes were the proposed strategies in this category (Figure).

**CDC category 4: strategies to encourage physical activity or limit sedentary activity among children and youth**

Fifteen of the 41 partnerships proposed strategies to encourage physical activity or limit sedentary activity among children and youth (Table 2). HKHC partnerships proposed strategies consistent with all the CDC recommendations in this category (Figure); the most common was increasing opportunities for physical activity outside of school. Ten partnerships proposed working collaboratively with schools to implement joint-use agreements that would expand use of school and community facilities for physical activity. HKHC partnerships proposing to establish standards for physical activity in after-school settings were classified under the CDC recommendation to increase the amount of physical activity in physical education programs in schools.

**CDC category 5: safe communities that support physical activity**

Thirty-five partnerships proposed strategies to create safe communities that support physical activity, making these the most frequently adopted set of strategies (Table 2). Improving access to outdoor recreation facilities and enhancing infrastructure to support walking and cycling were the most commonly proposed, followed by strategies to improve personal and traffic safety in areas where people could be physically active (Figure). Examples for improving access to outdoor recreation facilities included efforts to revitalize parks, enhance community awareness about such facilities, and improve their connectivity to residents. Safe Routes to School was mentioned by more than half of the partnerships working in this area. Sidewalks, bike lanes, improved connectivity between pedestrian and bike paths, traffic calming, and crossing aids were some ways partnerships proposed to improve infrastructure for promoting walking and cycling. To enhance personal and traffic safety, most efforts focused on increased police presence, street lighting, traffic calming patterns, and plans to design "complete" streets with all users in mind, including bicyclists, public transport vehicles and riders, and pedestrians.

### Additional common strategies proposed by HKHC communities

HKHC partnerships proposed additional strategies for preventing childhood obesity. Twenty-three partnerships proposed community gardens, making it 1 of the 2 most common community strategies (the other being to enhance infrastructure to support walking). Seven partnerships proposed creating local food policy councils. The focus of the call for proposals was on policy, systems, and environmental change approaches; health education, marketing, and other promotion strategies were acceptable as part of the overall plan, as long as other funding sources supported such efforts. Eleven partnerships proposed health promotion (eg, social marketing campaign) and education efforts (eg, educational videos, curriculum design).

### Which communities choose which strategies?

Of the 4 partnerships that proposed strategies to support healthy food and beverage choices, all were urban communities from priority states, 3 were in the lowest median household income group, 2 were large (population >250,000), and 2 were communities with low proportions of Hispanics and African Americans (Table 2). Partnerships that identified strategies to encourage physical activity and to limit sedentary behavior in children were also primarily urban, with lower proportions of Hispanics and African Americans. No other patterns based on community characteristics emerged.

## Discussion

The content analysis of 41 HKHC-funded proposals suggests that these community partnerships were already moving in several directions that CDC would recommend soon thereafter. Although RWJF was involved in both projects, the HKHC call for proposals was developed independently from the CDC recommendations. The way in which the HKHC proposals reflected the soon-to-be recommended strategies are therefore noteworthy and encouraging because it implies that the HKHC community partnerships perceive the CDC recommendations to be feasible. The communities could visualize and describe how they would implement such abstract concepts as "promote food access" to achieve their aims. In the absence of evidence for effectiveness, implementation decisions are often based on perceptions of impact, taking into consideration community assets and challenges (6).

Better access to food and recreation facilities and increased personal and traffic safety were common approaches among the 41 grantees. Their community partnerships could envision how to implement these environmental changes. Indeed, many examples of successful food-access efforts in other communities are now available (7,8). Moreover, cities, counties, municipalities, and neighborhoods often have the authority to affect the availability and location of healthy food and to support mechanisms promoting local foods. The same is true for improving access to facilities for physical activity, including cycling and walking, and for improving traffic and personal safety (9,10).

Some approaches, however, were not as common among proposals: influencing healthy food choices, limiting calorie-dense foods and beverages outside of school, and zoning for mixed-use development to encourage physical activity. Most examples of success in setting these standards and limits are from schools (11-14), and communities may perceive them as infeasible outside the school day. Additional technical assistance may be needed to translate these recommendations into practical, implementable activities.

HKHC partnerships proposed some strategies that CDC did not recommend. Foremost in that category are community gardens. Despite scant evidence for their role in improving nutrition (15) and in preventing childhood obesity, the popularity of community gardens in HKHC proposals likely results from the recognition that these gardens promote community involvement, neighborhood revitalization, and green and sustainable environments (16).

For the most part, the CDC-recommended strategies were applicable to the communities regardless of their size, demographic makeup, location, type of leadership, or age of their partnership. Community differences did appear, however, for the strategies to encourage physical activity and limit sedentary behavior. Urban communities and communities with the lowest proportions of African American and Hispanic populations were most likely to choose strategies to encourage physical activity and limit sedentary behavior. The greater probability of joint-use agreements between communities and schools in urban areas may be due to easier access to school and community facilities compared with access in communities with less dense populations. The lack of these strategies in proposals from predominantly African American and Hispanic communities is of concern. Environmental and policy change strategies show enormous potential, yet are often underused in populations with health disparities (17,18).

Our analysis has several limitations. The proposals were selected on the basis of criteria of community need (location, income levels, predominant race/ethnicity), and grants were awarded based on a selection committee's joint assessment of merit. Therefore, they are not necessarily representative of community plans for preventing childhood obesity in the United States. Abstracting strategies from the proposal narratives was challenging because applicants presented their plans in multiple places in their proposals. Every effort was made to abstract information, but some proposed strategies may have been overlooked. Lastly, abstracting and matching proposed strategies with CDC recommendations was a subjective process. To improve the reliability of abstraction, 2 coders matched the strategies and, in case of discrepancies, reached consensus through discussion with another author.

A comparison of strategies proposed by the HKHC-funded partnerships and CDC's recommendations for preventing obesity shows that the 2 overlap considerably. Areas for which CDC recommendations were more commonly proposed included providing incentives to food retailers to offer healthier options in underserved areas, increasing the number of farmers' markets, improving access to outdoor recreation facilities, enhancing cycling and walking infrastructure, and enhancing personal and traffic safety. The communities likely consider these strategies feasible to implement, especially given examples in the field. On the other hand, few strategies were proposed by HKHC partnerships in areas where previous implementation had been limited to schools. Changing environments and policies relevant during the school day was not the focus of HKHC. With the exception of joint-use agreements being more popular in urban areas and in areas with lower African American and Hispanic populations, use of strategies did not differ by community demographic characteristics.

In addition to providing a snapshot of communities' thinking on approaches for policy, systems, and environmental change strategies for preventing childhood obesity, our analysis provides an example of how information from communities, obtained through grant-writing efforts, can be used to assess the status of the field, guide future research, and provide direction for future investments.

## Figures and Tables

**Table 1:** Demographic Characteristics of Funded Sites, Healthy Kids, Healthy Communities Program, 2009

**Community Characteristic**	Communities in Priority States^a^(n = 23)	Communities in Nonpriority States(n = 18)
**Size of community**		
Small (<100,000)	10	7
Medium (100,000-249,999)	7	5
Large (≥250,000)	6	6
**Median annual household income, $**		
<25,000	5	2
25,000-49,999	18	14
50,000-75,000	0	2
**Administrative jurisdiction**		
Municipal	9	12
County	7	5
Region	4	1
Other	3	0
**Location**		
Mixed	2	3
Mostly rural	9	2
Mostly suburban	1	2
Mostly urban	11	11
**African American population, %**		
<25	12	11
25-50	7	5
>50	3	2
**Hispanic population, %**		
<25	16	13
25-50	3	2
>50	4	3
**Age of partnership, y**		
<1	7	4
1-2	4	7
3-5	5	3
>5	7	4

a States with a high prevalence of childhood obesity.

**Table 2:** Community Characteristics by Strategies Proposed by Healthy Kids, Healthy Communities (HKHC) Program that Matched CDC Recommendations for Preventing Childhood Obesity,^a^ 2009

Community Characteristic	Overall (n = 41)	CDC Category,^b^ No. of HKHC Sites Proposing Strategies			

		1: Promote Availability of Affordable Healthy Foods and Beverages (n = 31)	2: Support Healthy Food and Beverage Choices (n = 4)	4: Encourage Physical Activity or Limit Sedentary Behavior Among Children and Youth (n = 15)	5: Create Safe Communities That Support Physical Activity (n = 34)
**Size of community**					
Small (<100,000)	14	10	1	5	12
Medium (100,000-249,999)	15	13	1	7	12
Large (≥250,000)	12	8	2	3	10
**Priority state^c^**					
No	18	13	0	7	15
Yes	23	18	4	8	19
**Median annual household income, $^d^**					
<25,000	7	6	3	1	4
25,000-49,999	32	23	1	13	28
50,000-75,000	2	2	0	1	2
**Administrative jurisdiction**					
Municipal	21	14	2	7	15
County	12	9	1	3	9
Region	5	5	0	3	4
Other	3	3	1	2	6
**Location**					
Mixed	5	4	0	1	4
Mostly rural	11	7	0	2	8
Mostly suburban	3	1	0	2	2
Mostly urban	22	19	4	10	20
**African American population, %**					
<25	23	16	2	10	19
25-50	12	10	1	2	10
>50	5	4	1	2	4
**Hispanic population, %**					
<25	29	21	2	12	24
25-50	5	4	1	1	4
>50	7	6	1	2	6
**Age of partnership applying for funding, y**					
<1	11	9	0	6	4
1-2	11	11	3	3	3
3-5	8	5	0	4	4
>5	11	6	1	4	4
**Lead organization**					
Nonprofit	19	13	2	8	15
Education	5	5	0	1	4
Philanthropy	3	3	0	1	3
Government	14	10	2	5	12

Abbreviation: CDC, Centers for Disease Control and Prevention.

a Refers to CDC recommendations (3) that matched proposed strategies for grant submissions to the HKHC program.

b CDC category 3 was not used in this analysis because it refers to breastfeeding. CDC category 6 was not used because it refers to community coalitions, which existed for all HKHC grantees.

c Communities from states with a high prevalence of childhood obesity.

d Median household income as reported by communities.
