# Erratum, Vol. 9, Dec. 15 Release

**Published:** 2012-01-12

In the article “Strategies Proposed by Healthy Kids, Healthy Communities Partnerships to Prevent Childhood Obesity,” an author’s name was misspelled. The correct spelling is Laura K. Brennan. The correction was made to our website on January 12, 2012, and appears online at http://www.cdc.gov/pcd/issues/2012/10_0292.htm. We regret any confusion or inconvenience this error may have caused.

